# Inhibition of glycogen synthase kinase-3 by BTA-EG_4_ reduces tau abnormalities in an organotypic brain slice culture model of Alzheimer’s disease

**DOI:** 10.1038/s41598-017-07906-1

**Published:** 2017-08-07

**Authors:** Cara L. Croft, Ksenia Kurbatskaya, Diane P. Hanger, Wendy Noble

**Affiliations:** 1 Department of Basic and Clinical Neuroscience, King’s College London, Institute of Psychiatry, Psychology & Neuroscience, Maurice Wohl Clinical Neuroscience Institute, London, SE5 9RX UK; 2 Department of Neuroscience, University of Florida, Gainesville, Florida 32610 USA

## Abstract

Organotypic brain slice culture models provide an alternative to early stage *in vivo* studies as an integrated tissue system that can recapitulate key disease features, thereby providing an excellent platform for drug screening. We recently described a novel organotypic 3xTg-AD mouse brain slice culture model with key Alzheimer’s disease-like changes. We now highlight the potential of this model for testing disease-modifying agents and show that results obtained following *in vivo* treatment are replicated in brain slice cultures from 3xTg-AD mice. Moreover, we describe novel effects of the amyloid-binding tetra (ethylene glycol) derivative of benzothiazole aniline, BTA-EG_4_, on tau. BTA-EG_4_ significantly reduced tau phosphorylation in the absence of any changes in the amounts of amyloid precursor protein, amyloid-β or synaptic proteins. The reduction in tau phosphorylation was associated with inactivation of the Alzheimer’s disease-relevant major tau kinase, GSK-3. These findings highlight the utility of 3xTg-AD brain slice cultures as a rapid and reliable *in vitro* method for drug screening prior to *in vivo* testing. Furthermore, we demonstrate novel tau-directed effects of BTA-EG_4_ that are likely related to the ability of this agent to inactivate GSK-3. Our findings support the further exploration of BTA-EG_4_ as a candidate therapeutic for Alzheimer’s disease.

## Introduction

Alzheimer’s disease (AD) is characterised pathologically by the presence, predominantly in the hippocampus, neocortex and interconnecting regions, of β-amyloid (Aβ)-containing extracellular plaques and intracellular neurofibrillary tangles comprising hyperphosphorylated and cleaved forms of tau^1–3^. Associated with the development and progression of AD are synaptic and neuronal dysfunction, widespread synaptic and neuronal loss, upregulation of proteolytic calpains and caspases, calcium dyshomeostasis, altered protein kinase activities, increased oxidative damage and activation of inflammatory cascades, all of which contribute to cognitive decline and other clinical symptoms of AD^4–6^. There are currently no disease-modifying treatments for AD, despite intensive testing of potential new therapies^7, 8^.

BTA-EG_4_ is an amyloid-binding drug which reduces Aβ-induced toxicity *in vitro* ^9^. BTA-EG_4_ readily crosses the blood-brain-barrier and is soluble in aqueous environments^10^. It has previously been shown to reduce production of Aβ-40, and increase synaptic density and function in wild-type (WT) mice *in vivo* ^11^. In addition, treatment of early disease-stage 3xTg-AD mice with BTA-EG_4_ was found to increase dendritic spine density, drive other alterations in spine morphology reflective of recovered synaptic function, and improve cognitive performance^12^. Tau-associated synaptotoxicity is also prevalent in AD^13, 14^, yet the effects of BTA-EG_4_ on tau have not been explored. Furthermore, despite its reported beneficial effects, little is known about the mode of action of BTA-EG_4._

Typically, identification of compounds to target dementia occurs in transgenic mouse models expressing genes that cause familial forms of this disease. Novel methods that provide more rapid screening of drug candidates to identify lead compounds prior to their pre-clinical testing *in vivo* would be welcomed by the field^15^. We have recently shown that some prominent molecular phenotypes of neurodegeneration exhibited by 3xTg-AD mice are recapitulated in organotypic brain slice cultures produced from these mice at postnatal day 8–9 and subsequently maintained in culture for up to 28 days *in vitro* (DIV)^16^. 3xTg-AD brain slice cultures rapidly show increased production of Aβ-42, an increased Aβ-42/Aβ-40 ratio, tau phosphorylation at AD-relevant epitopes, such as Ser202 and Ser396/404, and tau mislocalisation and altered release when compared to control WT slice cultures^16^. Importantly, these molecular phenotypes are accelerated in culture compared to *in vivo*. The work presented here extends these findings and highlights 3xTg-AD brain slice cultures as a sensitive *ex vivo* model with a high potential for AD drug discovery.

We found that treating 3xTg-AD slice cultures with the GSK-3 inhibitor lithium chloride (LiCl), or the microtubule-binding agent NAPVSIPQ, reduces tau phosphorylation at sites implicated in AD, recapitulating previously reported *in vivo* findings in aged 3xTg-AD mice^17–19^. We also present novel data showing tau-directed effects of BTA-EG_4_ that are associated with BTA-EG_4_-mediated inhibition of GSK-3. These data highlight the utility of organotypic brain slice culture models for accelerated drug screening and support further exploration of BTA-EG_4_ and related derivatives for the treatment of AD and other tauopathies.

## Results

### Treatment of organotypic brain slice cultures from 3xTg-AD mice with LiCl and NAPVSIPQ recapitulates *in vivo* effects on tau phosphorylation

We have recently reported that organotypic brain slice cultures from 3xTg-AD postnatal day 8–9 mice and maintained in culture for up to 28 DIV develop key AD-like molecular features^16^ which recapitulate the *in vivo* degenerative phenotype observed in 3xTg-AD mice^20, 21^. These include the progressive accumulation of hyperphosphorylated tau, tau mislocalisation and altered tau release rates, altered APP processing and Aβ-42 accumulation^16^. To validate the use of this *ex vivo* slice culture model for drug discovery, we first treated the cultures with compounds previously shown to reduce tau phosphorylation in 3xTg-AD *in vivo*.

**Figure 1:**
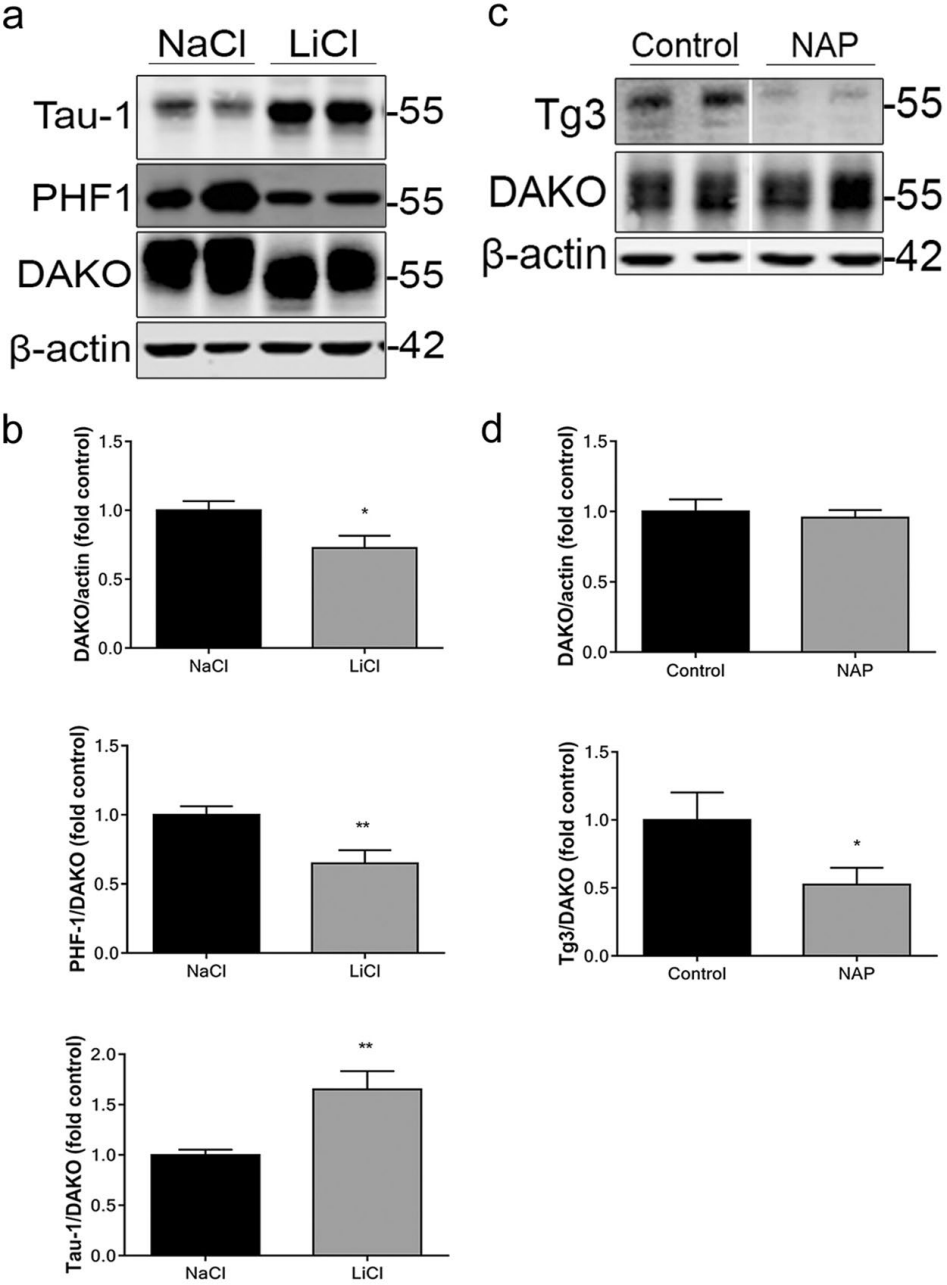
LiCl and NAPVSIPQ reduces tau phosphorylation in organotypic brain slice cultures from 3xTg-AD mice. Representative western blots of lysates from 28 DIV 3xTg-AD organotypic brain slice cultures treated with (**a**) 20 mM LiCl or 20 mM NaCl (control) for 4 h, or (**c**) 100 nM NAP or control (H_2_O) for 4 h. Blots were probed with antibodies against total tau (both non-phosphorylated and phosphorylated; DAKO), PHF-1 (phospho-Ser396/404), Tau-1 (dephospho-Ser202/Thr205) and Tg3 (phospho-Thr231). Blots were also probed with an antibody against β-actin as a loading control. Bar charts show amounts of total tau relative to β-actin in each sample, and phospho-tau as a proportion of total tau in each sample after treatment with (**b**) 20 mM LiCl or 20 mM NaCl (control) (all data analysed by unpaired t-test) or (**d**) 100 nM NAP or control (H_2_O). n = 9–12. (DAKO/actin analysed by Mann Whitney U test, Tg3/DAKO analysed by unpaired t-test). Data is mean ± SEM and is shown as fold change from control. *p < 0.05, **p < 0.01.

The therapeutic potential of the GSK-3 inhibitor LiCl has been widely explored in various models of AD^22–25^. Notably, LiCl treatment of 3xTg-AD mice significantly reduces tau phosphorylation at several epitopes that are abnormally phosphorylated in AD brain, including Thr181, Ser202/Thr205 and Thr231^17^. To examine the effects of LiCl in the 3xTg-AD *ex vivo* model, organotypic brain slice cultures were prepared from 3xTg-AD mice and aged for 28 DIV prior to treatment with 20 mM LiCl for 4 h. This dose has previously been shown to significantly reduce tau phosphorylation at several epitopes in primary neuronal cultures^26^, and does not affect neuronal viability (supplementary data). Changes in the total amount of tau and tau phosphorylation were assessed by immunoblotting (Fig. 1a). LiCl significantly reduced tau phosphorylation at the PHF-1 (Ser396/404) epitope, whilst also reducing the amount of total tau (Fig. 1b) compared to control (NaCl). LiCl also increased the amount of tau dephosphorylated at Ser202/Thr205 relative to total tau, detected using the Tau-1 antibody. There was also a notable shift in the apparent molecular weight of tau in lysates from LiCl treated slice cultures, which is characteristic of reduced tau phosphorylation^27^. Thus, treatment of 3xTg-AD slice cultures with LiCl mimics the reduction in tau phosphorylation observed following treatment of 3xTg-AD mice *in vivo*.

NAPVSIPQ (NAP) is a neuroprotective peptide which has previously been shown to reduce tau phosphorylation *in vitro* and *in vivo* ^18, 19, 28, 29^. Treatment of 3xTg-AD mice with NAP was shown to significantly reduce tau phosphorylation at Thr231^18, 19^, a site important for tau binding to microtubules^30^. Slice cultures prepared from 3xTg-AD mice and aged for 28 DIV were treated with 100 nM NAP for 24 h prior to analysis of changes in tau amounts and phosphorylation at Thr231 (Fig. 1c). This concentration of NAP had previously shown efficacy to reduce tau phosphorylation in cultured neural cells^28^, without affecting neuronal viability (supplementary data). NAP treatment of 3xTg-AD slice cultures significantly reduced tau phosphorylation at the Tg3 (Thr231) epitope, but did not alter the total amount of tau when compared to control cultures (Fig. 1d). These data further confirm that the effects of tau-directed treatments *in vivo* can be recapitulated in *ex vivo* slice cultures that model human disease.

### Treatment of 3xTg-AD slice cultures with BTA-EG_4_ reduces tau phosphorylation but does not alter the amount of Aβ

**Figure 2:**
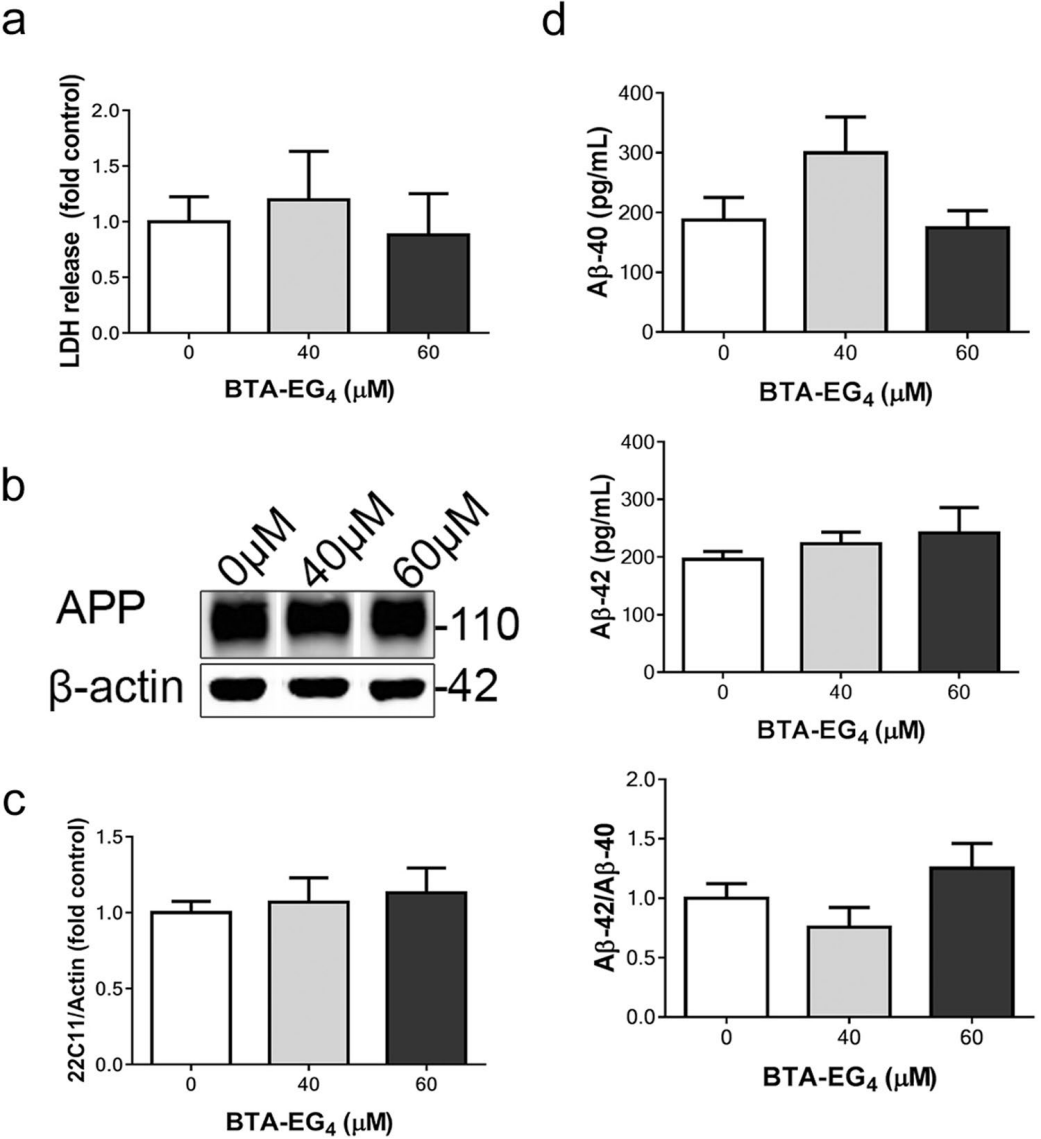
BTA-EG_4_ treatment of 3xTg-AD brain slice cultures does not alter APP processing, amyloid load or cell viability. (**a**) Bar chart shows the proportion of LDH released as a proportion of total LDH following treatment of 3xTg-AD slice cultures with control (DMSO, 0 μM), 40 μM or 60 μM BTA-EG_4_ for 48 h. Data is mean ± SEM and is shown as fold change from control. n = 12. (**b**) Representative western blots of lysates prepared from 3xTg-AD slice cultures treated with 40–60 μM BTA-EG_4_ and control (DMSO, 0 μM) for 48 h probed with an antibody against N-terminal APP (22C11) and β-actin as a loading control. (**b**) Bar chart shows amounts of total APP relative to β-actin amounts in the same sample. n = 12. Data is mean ± SEM and is shown as fold change from control. (**c**) Amounts of Aβ-40 and Aβ-42 were measured in BTA-EG_4_-treated slice cultures using specific Aβ-40 and Aβ-42 ELISAs. Bar charts show amounts of Aβ-42 and Aβ-40. Data are shown in pg/mL. The ratio of Aβ-42 relative to Aβ-40 is also shown. Data is mean ± SEM and are shown as fold control. n = 4. All data analysed by one-way ANOVA with post-hoc Dunnett’s multiple comparisons test.

The effects of the amyloid-binding agent BTA-EG_4_ were next explored in 3xTg-AD slice cultures. It was first important to establish an effective dose for treatment by determining any toxicity resulting from 48 h treatment of 28 DIV 3xTg-AD cultures with BTA-EG_4_. Therefore, lactate dehydrogenase (LDH) release into culture medium, was calculated as a proportion of total LDH following treatment of slices with 40 μM or 60 μM BTA-EG_4_. Neither concentration of BTA-EG_4_ significantly affected total LDH release, indicating that BTA-EG_4_ does not cause toxicity in cultured brain slices at these concentrations (Fig. 2a). This dose was also not toxic to primary cultured neurons (supplementary data). This dose had previously been shown to be cytoprotective *in vitro* against Aβ toxicity in SH-SY5Y cells in a 24 h treatment period^9^

Previous reports have suggested that *in vivo* treatment with BTA-EG_4_ increases sAPPα and reduces sAPPβ, leading to reduced Aβ40 in the brains of WT mice^11^. BTA-EG_4_ also protects against Aβ toxicity and prevents the interaction of amyloid fibrils with amyloid-binding proteins *in vitro* ^9, 10^. It was therefore important to determine whether BTA-EG_4_ modified APP and/or Aβ in 3xTg-AD slices, since these show altered APP processing and accumulation of Aβ-42^16^. Treatment with 40 or 60 μM BTA-EG_4_ did not affect levels of N-terminal APP, as measured by the 22c11 antibody, in comparison to controls (Fig. 2b,c), which is in keeping with previous findings in WT mice^11^.

3xTg-AD slice cultures produce increased amounts of Aβ-42 resulting in an altered Aβ-42/40 ratio^16^. However, a limitation of this system is that Aβ does not appear to deposit or aggregate in these slice cultures by 28 DIV. However, since Megill *et al*.^11^ reported that BTA-EG_4_ reduces the amount of Aβ-40 in the brains of WT mice, it was important to determine the effects of BTA-EG_4_ on Aβ levels in 3xTg-AD cultures. Aβ-42 and Aβ-40 amounts in treated and control (DMSO) 3xTg-AD slice cultures were measured by ELISA, as previously described^16^. BTA-EG_4_ had no effect, compared to control, on amounts of Aβ-42, Aβ-40 or the Aβ-42/Aβ-40 ratio in 3xTg-AD brain slice cultures (Fig. 2d).

**Figure 3:**
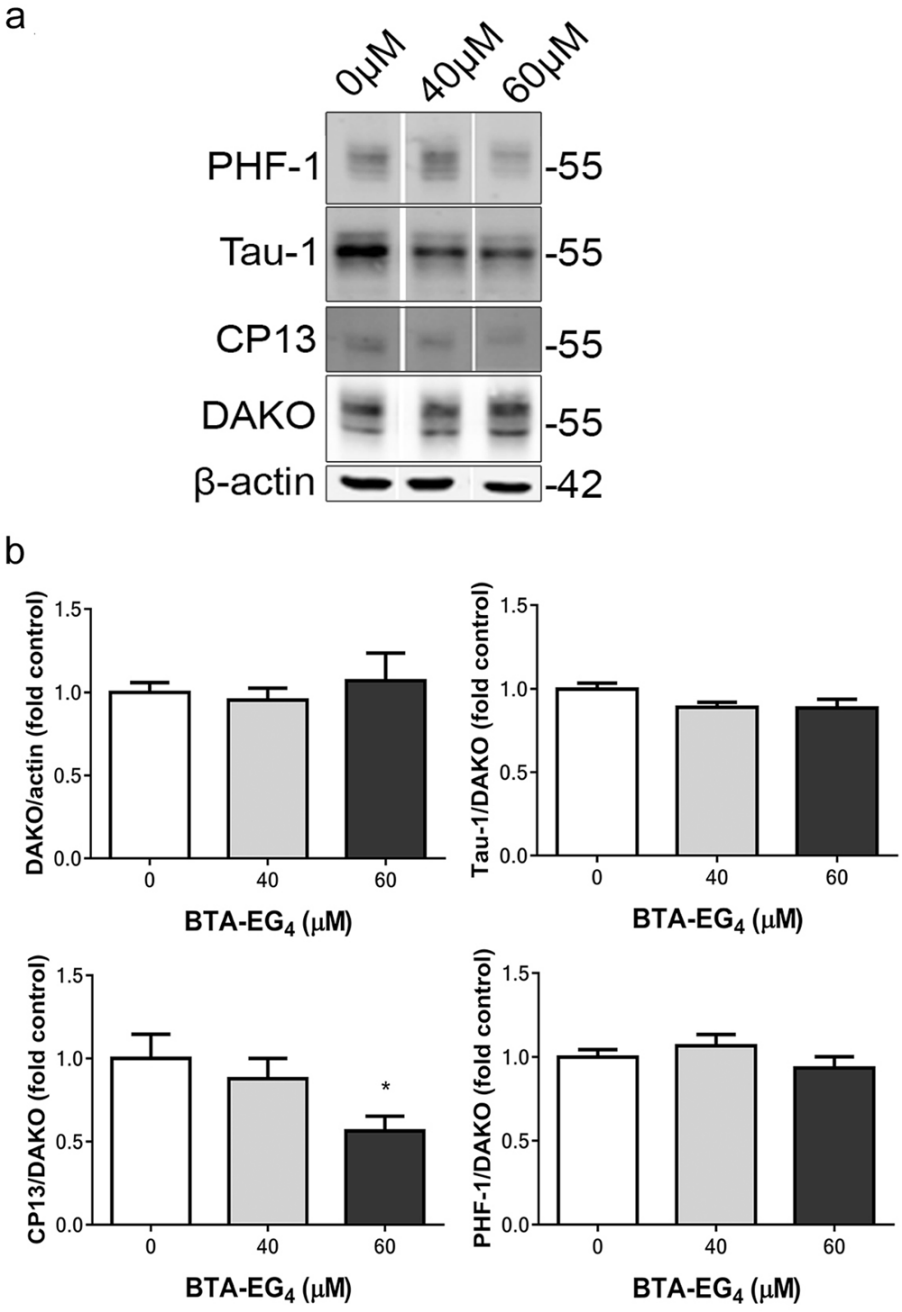
BTA-EG_4_ reduces tau phosphorylation in 3xTg-AD brain slice cultures. Representative western blots of lysates from 28 DIV BTA-EG_4_-treated and control (vehicle, DMSO)-treated 3xTg-AD slice cultures. Blots were probed with antibodies against total tau (both non-phosphorylated and phosphorylated; DAKO), PHF-1 (phospho-Ser396/404), Tau-1 (dephospho-Ser199/202/Thr205) and CP13 (phospho-Ser202). Blots were also probed with an antibody against β-actin as a loading control. (C) Bar charts show amounts of total tau relative to β-actin in each sample, and phospho-tau as a proportion of total tau in each sample after treatment with BTA-EG_4_ or control. n = 12. Data is mean ± SEM and is shown as fold change from control. *p < 0.05. All data analysed by one-way ANOVA, with post-hoc Dunnett’s multiple comparisons test.

**Figure 4:**
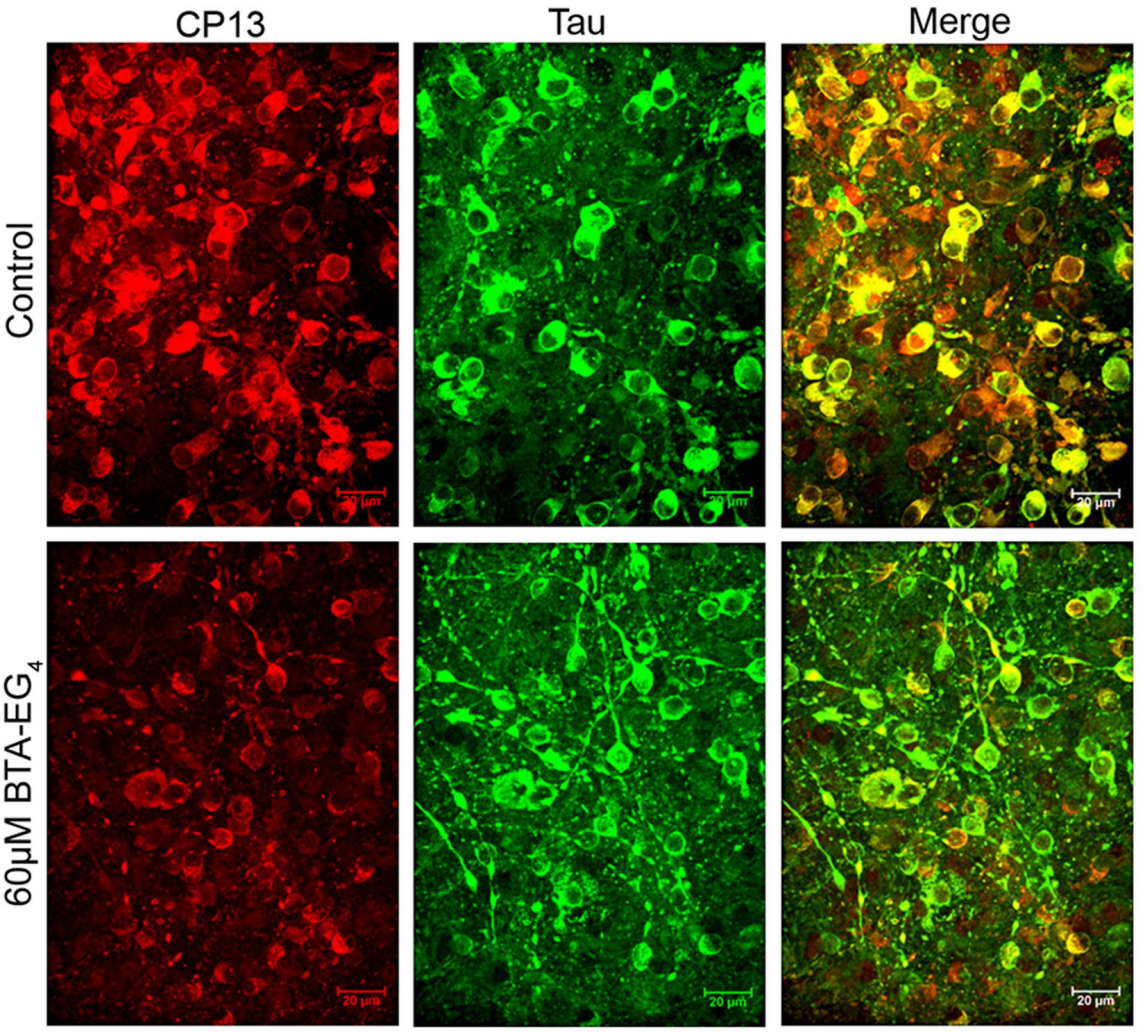
BTA-EG_4_ reduces tau phosphorylation and increases axonal tau distribution in 3xTg-AD brain slice cultures. Representative images from fixed 3xTg-AD organotypic brain slice cultures cultured for 28 DIV and treated with control (DMSO, 0 μM) or 60 μM BTA-EG4 for 48 h, immunolabelled with antibodies against total tau (both non-phosphorylated and phosphorylated; DAKO) - green and CP13 (phospho-Ser202) - red. Scale bar is 20 μm.

The effects of BTA-EG_4_ on tau amounts and phosphorylation were next examined by immunoblotting. Treatment of 3xTg-AD slices for 48 h treatment with 40 or 60 μM BTA-EG_4_ did not affect the amount of total tau, tau dephosphorylated at Ser199/202/Thr205 (Tau-1), or tau phosphorylated at Ser396/404 (PHF-1), relative to controls (Fig. 3a,b). In contrast, the amounts of tau phosphorylated at Ser202 (CP13) in 3xTg-AD slices were significantly reduced following 60 μM BTA-EG_4_ treatment when compared to controls (Fig. 3a,b). This significant reduction in tau phosphorylated at the CP13 epitope was confirmed upon immunofluorescent labelling of fixed brain slice cultures (Fig. 4). It is also apparent from immunolabelling that tau is redistributed from cell soma into axons upon BTA-EG_4_ treatment (Fig. 4). These findings illustrate novel tau-directed effects of BTA-EG_4_.

### BTA-EG_4_ does not alter synaptic proteins in 3xTg-AD brain slice cultures

**Figure 5:**
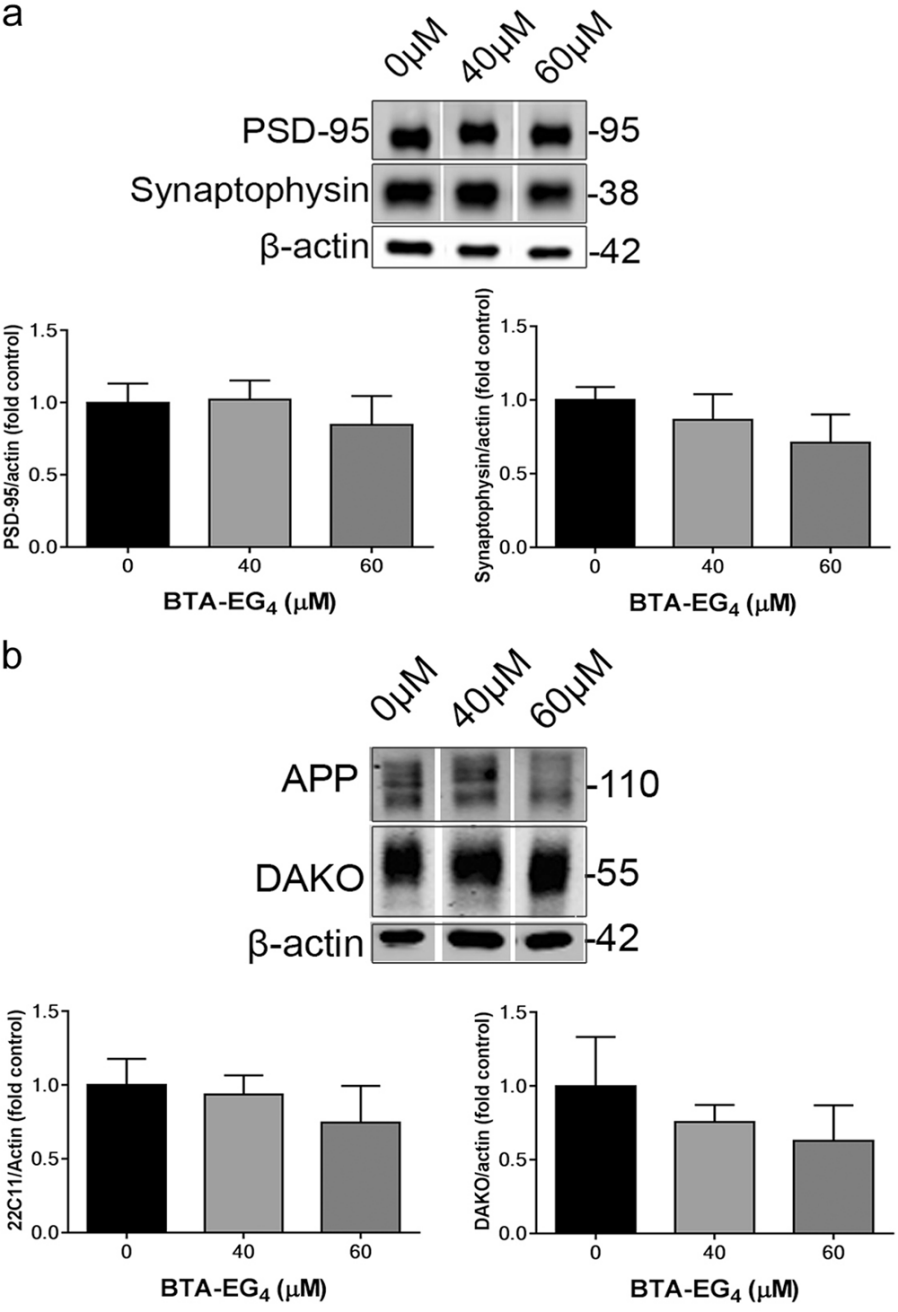
Levels of synaptic markers and synaptic APP and tau are unaltered following BTA-EG_4_ treatment. Representative western blots of synaptosome fractions isolated following 48 h 40–60 μM BTA-EG_4_ and control (DMSO, 0 μM) treatment of 3xTg-AD slice cultures. Blots are probed with antibodies against (**a**) synaptophysin and PSD-95 and (**b**) N-terminal APP (22C11) and total tau (phosphorylated and non-phosphorylated; DAKO). Blots were also probed with an antibody against β-actin as a loading control. Bar charts show amounts of (**a**) PSD-95 or synaptophysin, and (**b**) N-terminal APP (22C11) or tau (DAKO) relative to β-actin levels in each sample. n = 5–10. Data is mean ± SEM shown as fold change from control. All data analysed by one-way ANOVA, with post-hoc Dunnett’s multiple comparisons test.

Synaptic dysfunction is a major correlate of early cognitive symptoms and dementia in AD^31–33^. It has previously been demonstrated that BTA-EG_4_ increases synapse density in WT and 3xTg-AD mice^11, 12^. We therefore sought to determine the effect of BTA-EG_4_ treatment on synaptic protein amounts in 3xTg-AD slices. Following treatment with BTA-EG_4_ or vehicle, synaptosomes were isolated from 28 DIV 3xTg-AD slice cultures and immunoblotted for pre-synaptic synaptophysin and post-synaptic density (PSD)-95 protein. We have previously demonstrated the stringency of this fractionation^34^. BTA-EG_4_ did not affect the amounts of either synaptophysin or PSD-95, relative to actin, suggesting that BTA-EG_4_ does not affect either pre-synaptic or post-synaptic integrity in 3xTg-AD slices (Fig. 5a).

We previously reported an early accumulation of tau and APP in the synaptic compartment in young 3xTg-AD mice, with similar changes in tau observed in 14 DIV 3xTg-AD slice cultures^16^. We therefore examined the effect of BTA-EG_4_ treatment on levels of APP and tau in synaptosomes from 3xTg-AD slice cultures. BTA-EG_4_ did not affect the amount of tau or APP at the synapse when compared to controls (Fig. 5b). These results are in-keeping with other reports that indicate highly localised effects of Aβ on tau kinases and phosphorylation^35^. Overall, these results indicate that BTA-EG_4_ causes reductions in tau phosphorylation at specific sites of relevance to AD in aged 3xTg-AD brain slice cultures, and that these changes in phosphorylation do not appear to be related to changes in Aβ production or tau localisation.

### BTA-EG_4_ inhibits GSK-3 to reduce tau phosphorylation

BTA-EG_4_ has been previously reported to bind amyloid and increase Ras-mediated spinogenesis;^9–11^ however, its mechanisms of action are largely unknown. Since we have identified an effect of BTA-EG_4_ on tau phosphorylation, it was of interest to begin to examine the pathways by which this effect might occur. Increases in tau phosphorylation in AD are thought to be driven primarily by imbalances in kinases and phosphatases, including in two major tau kinases; GSK-3 and Cdk5^3, 36, 37^. The site at which BTA-EG_4_ reduced tau phosphorylation in 3xTg-AD slice cultures (Ser202, recognised by antibody CP13) is a known target of both GSK-3 and Cdk5^38, 39^, therefore we investigated the effect of BTA-EG_4_ on the activities of these kinases.

**Figure 6:**
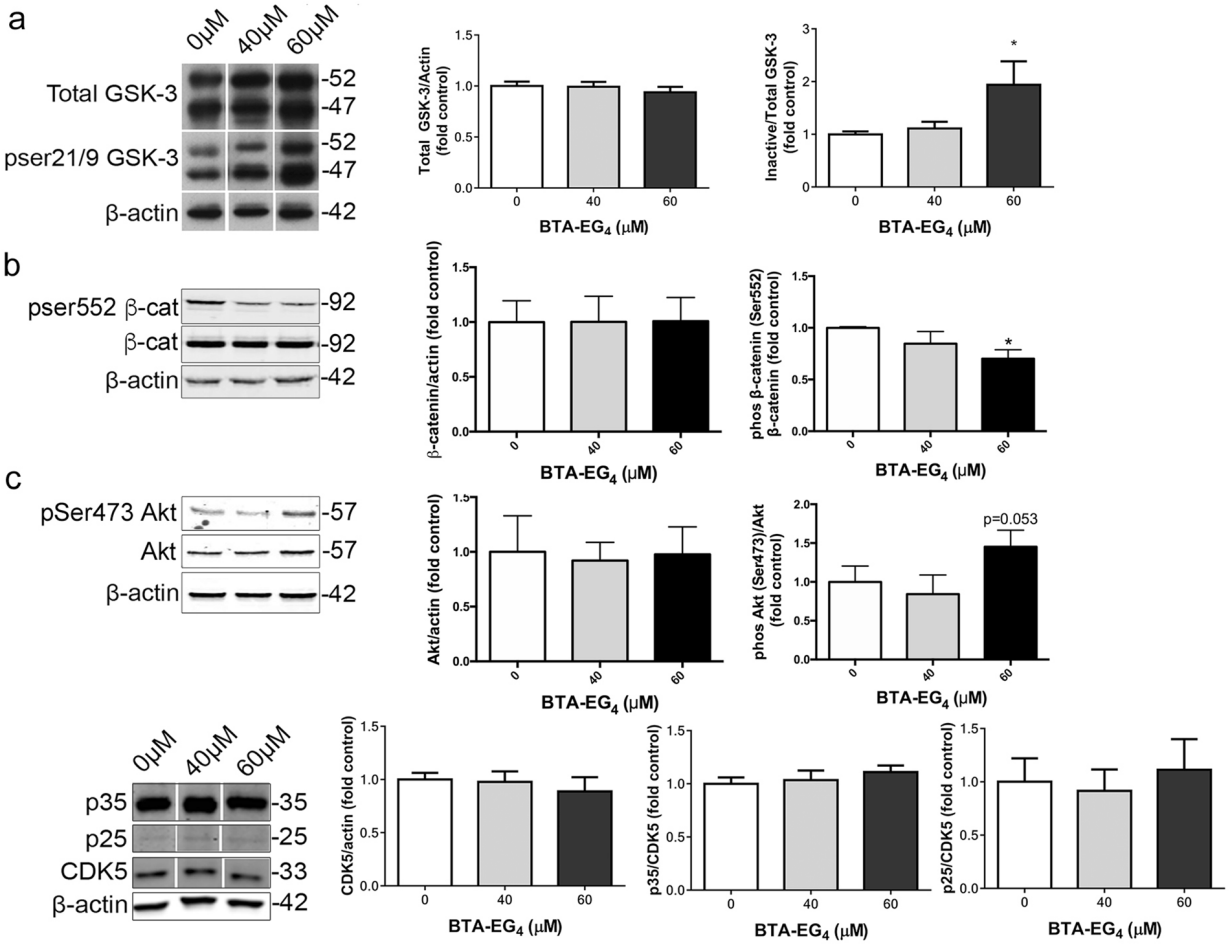
BTA-EG_4_ inhibits GSK-3 but not Cdk5 in 3xTg-AD slice cultures. Representative western blots of lysates following 48 h 40–60 μM BTA-EG_4_ and control (DMSO, 0 μM) treatment of 3xTg-AD slice cultures. (**a**) Blots were probed with antibodies against total GSK-3α/β and inactive GSK-3α/β (phosphorylated at ser 21/9). Bar chart shows amounts of total GSK-3 relative to β-actin and GSK-3 phosphorylated at ser 21/9 (inactive) as a proportion of total GSK-3, n = 12. (**b**) Western blots of β-catenin and β-catenin phosphorylated at ser 552, total Akt and active Akt (phosphorylated at ser 473), together with β-actin as a loading control. Bar charts show p-β-catenin relative to total β-catenin and β-catenin following normalisation to β-actin, and p-Akt relative to Akt and total Akt following normalisation to β-actin, n = 3. (**c**) Samples were immunoblotted with antibodies against cdk5, p35 (which also detects p25) β-actin. Bar charts show total Cdk5 relative to β-actin and p35/p25 as a proportion of Cdk5 in each sample. n = 12. All data is mean ± SEM and are shown as fold change from control. *p < 0.05. All data analysed by one-way ANOVA, with post-hoc Dunnett’s multiple comparisons test.

GSK-3α/β is predominantly regulated by inhibitory phosphorylation of Ser21/9, which causes inactivation of GSK-3 and downstream reductions in tau phosphorylation^24, 40, 41^. Levels of total and inactive (phosphorylated Ser21/9) GSK-3 were determined by immunoblotting of BTA-EG_4_-treated 3xTg-AD slice cultures (Fig. 6a). Total amounts of GSK-3 were unaffected by treatment with BTA-EG_4_; however, phosphorylation of GSK-3 at Ser21/9 was significantly increased by 60 μM, but not 40 μM, BTA-EG_4,_ relative to controls (Fig. 6a, p < 0.05). These data suggest that BTA-EG_4_ inhibits GSK-3 activity in a dose-dependent manner that likely results in the reduced phosphorylation of tau at Ser202 observed with this treatment. In support of this finding, similar dose-dependent decreases in the phosphorylation of another GSK-3 substrate, β-catenin^24, 42^, were observed upon BTA-EG_4_ treatment of slices in the absence of changes in total β-catenin amounts (Fig. 6b). To further investigate the mechanism linking BTA-EG_4_ with GSK-3, western blots were probed with antibodies against Akt and p-Akt. Activation of the Akt/protein kinase B pathway by phosphorylation of Akt at sites including Ser473 allows increased inhibitory phosphorylation of GSK-3α/β at Ser-9^24, 43^. These data show a trend towards significantly increased phosphorylation of Akt at Ser473 relative to total Akt upon 60 μM but not 40 μM BTA-EG4 treatment (Fig. 6b) suggesting that BTA-EG_4_ likely inhibits GSK-3 activity in an Akt-dependent manner.

Cdk5 activity is also known to drive phosphorylation of tau at Ser202^39, 44^. Cdk5 activity is predominantly regulated by its neuronal activators p35 and p25^45–47^, under physiological and pathological conditions, respectively^48^. Levels of total Cdk5 and amounts of p35 and p25 were analysed by immunoblotting (Fig. 6c) and were all found to be unaffected by BTA-EG_4_ (Fig. 6c), suggesting that the reductions in tau phosphorylation at Ser202 observed here are independent of Cdk5 and are likely to be mediated predominantly by inhibition of GSK-3 activity.

## Discussion

We show here novel tau-directed effects of the amyloid-binding agent BTA-EG_4_ that are associated with its inhibition of GSK-3 activity. These data support the view that 3xTg-AD organotypic brain slice cultures provide a novel and reliable model for AD drug discovery.

We first validated 3xTg-AD slice cultures as a model for exploring drug effects by demonstrating that LiCl and NAP reduce tau phosphorylation and recapitulate the effects of their administration on tau *in vivo* ^17, 18^. Importantly, the effects of these treatments were demonstrated here in 28 DIV cultures prepared from postnatal mouse pups, in contrast to prolonged treatment of adult 3xTg-AD mice. This highlights the notion that slice cultures can be used to provide an *ex vivo* model of the effects of AD-modifying treatments that is more time and cost-effective than currently used *in vivo* models, whilst significantly reducing the number of animals used and their aging to generate harmful phenotypes. Indeed, acute treatment of 3xTg-AD slice cultures with LiCl and NAP predicts changes in tau phosphorylation which have previously been confirmed with chronic treatment *in vivo* ^17, 18^.

Based on these findings it is plausible that slice cultures can be screened for candidates which show efficacy over acute periods of time and these could then be extrapolated to a more chronic treatment *in vivo*.

We next explored the use of BTA-EG_4_ on AD-like molecular changes in these slice cultures. We have shown that treatment of 3xTg-AD slice cultures with BTA-EG_4_ reduces phosphorylation of tau at Ser202 in association with GSK-3 inhibition that is mediated by increased GSK-3 phosphorylation at Ser21/9. Changes were also observed in Akt phosphorylation at Ser473, one of the sites implicated in Akt activation^49^, suggesting that the effects on GSK-3 are mediated by Akt. Phosphorylation of tau at Ser202 is increased in AD brain^50^, and GSK-3 is a known kinase for this site^44, 51^. Tau is a major target for drug discovery in AD^52^ since tau accumulation is most closely associated with dementia in AD^33^, and tau is necessary for Aβ-induced neuronal loss^13, 53^ and deficits in long-term potentiation^54^. Therefore, this study supports further exploration of BTA-EG_4_ as a potential AD therapeutic agent. We speculate based upon our findings in 3xTg-AD slice cultures that chronic BTA-EG_4_ treatment *in vivo* in these mice would also reduce tau phosphorylation mediated by GSK-3 inhibition, highlighting the predictive value slice culture systems can offer.

BTA-EG_4_ had previously been reported to have protective effects at the synapse in both WT and 3xTg-AD mice^11, 12^. In the slice culture system described here, no changes in levels of the pre-synaptic and post-synaptic markers, synaptophsyin and PSD-95, were observed upon treatment with BTA-EG_4_. This is likely due to the fact that 3xTg-AD slice cultures show no overt synapse loss at 28 DIV^16^. Alternatively, it has been suggested that slice cultures may develop stronger synaptic connectivity than *in vivo* and thus are less susceptible to synapse loss^55^.

BTA-EG_4_ increased the amount of sAPPα and reduced sAPPβ without affecting the total amount of APP in WT mice^11^, suggesting an ability of BTA-EG_4_ to alter APP processing. In agreement with this, we found no changes in the total amount of APP, and an apparent increase in mature APP. Due to the low levels of secreted sAPPα and sAPPβ, we were unable to measure these components in this study. In contrast to the findings of Megill *et al*.^11^ and Song *et al*.^12^; however, who treated WT and 3xTg-AD mice with BTA-EG_4_, respectively, we did not detect any changes in Aβ-40, nor did we observe any effects on Aβ-42 or the Aβ-42/Aβ-40 ratio. However, the reducing effect of BTA-EG_4_ treatment on Aβ-40 was lost in older 3xTg-AD mice^12^ and it is possible that the apparent accelerated aging that occurs in 3xTg-AD slices^16^, may prevent any effect of BTA-EG_4_ effect on Aβ abundance in 3xTg-AD cultures. This finding further emphasises the critical importance of taking into account the disease stage when investigating potential AD-modifying treatments. It is also conceivable that in the 3xTg-AD slice cultures, BTA-EG_4_ is more potent towards reducing tau phosphorylation through GSK-3 inhibition compared to Aβ reduction likely by alternative mechanisms. With this in mind, we propose that BTA-EG_4_ is an effective GSK-3 inhibitor which potently reduces tau phosphorylation in a 3xTg-AD slice culture system. It is important to confirm these findings *in vivo*, but this adds to previous findings of others which show that BTA-EG_4_ is protective against Aβ and is synaptotrophic *in vivo* ^11, 12^ highlighting the potential of BTA-EG_4_ to target three major features of AD.

To conclude, we have highlighted the utility of 3xTg-AD brain slice cultures for AD drug discovery and development. We show the value of this system for identifying molecular changes relevant for investigating new AD treatments in a significantly shorter timescale than exists in *in vivo* paradigms. We have also identified novel tau-directed effects of BTA-EG_4_ that are associated with inhibition of GSK-3. Taken together, these findings support further investigation into the potential beneficial effects of BTA-EG_4_ and similar compounds for AD and related tauopathies.

## Methods and Materials

All materials were obtained from Sigma (Poole, Dorset, UK) unless otherwise stated.

### Mice and preparation of organotypic brain slice cultures

3xTg-AD^20, 21^ mice were used in this study. All methods were carried out in accordance with the UK Animals (Scientific Procedures) Act 1986. All experiments with mice were conducted under UK Home Office Personal and Project Licenses and with agreement from the King’s College London (Denmark Hill) Animal Welfare and Ethical Review Board.

Slice cultures were prepared from postnatal day 8–9 3xTg-AD^20, 21^ mice, as previously described^16^. In brief, pups were decapitated and the brains removed and dissected to retain the cortex, hippocampus and connecting regions in each hemi-brain in sterile filtered ice-cold dissection buffer (1.25 mM KH_2_PO_4_, 124 mM NaCl (pH 7.4), 3 mM KCl, 8.19 mM MgSO_4_, 2.65 mM CaCl_2_, 3.5 mM NaHCO_3_, 10 mM glucose, 2 mM ascorbic acid, 39.4 μM ATP in ultrapure H_2_O). The hemi-brain was placed on filter paper and 350 µm coronal slices were cut using a McIllwain^TM^ tissue chopper (Mickle Laboratory Engineering Co. Ltd., Surrey, UK). The 18, 350 μM slices were collected and plated in sequence; three consecutive slices per semi-porous membrane insert (Millipore, 0.4 µm pore diameter, Fisher Scientific, Loughborough, UK) in 6-well sterile culture plates. Slices from the same part of the brain are therefore in the same wells of each plate. For treatments; the same wells in each plate were treated with vehicle or drug allowing comparison of drug effects in the same brain areas. Slices were maintained at 37 °C and 5% CO_2_ in sterile-filtered culture medium containing basal medium eagle (BME), 26.6 mM HEPES (pH 7.1), 19.3 mM NaCl, 5 mM NaHCO_3_, 511 μM ascorbic acid, 40 mM glucose, 2.7 mM CaCl_2_, 2.5 mM MgSO_4_, 1% (v/v) GlutaMAX (Life Technologies, Paisley, UK), 0.033% (v/v) insulin, 50 U/ml penicillin, 50 μg/ml streptomycin and 25% (v/v) heat-inactivated horse serum. Culture medium was changed every 2–3 days. Slice cultures were treated after 28 days *in vitro* with 40–60 μM BTA-EG_4_ for 48 h, 20 mM LiCl for 4 h, 100 nM NAP (Alpha Diagnostic International, TX, USA) for 24 h, and with the appropriate vehicles.

### Cytotoxicity assays

Cell death in slice cultures was evaluated by measuring LDH in culture medium using Cytotox 96 assay kits (Promega, Madison, WI, USA), according to the manufacturer’s protocol. Optical density was measured at 492 nm (Wallac 1420 Victor^3^ plate reader, PerkinElmer, Waltham, MA, USA). LDH content in medium and lysed slice cultures was summed to determine total LDH content. LDH release from slice cultures was calculated as a percentage of total LDH (LDH in medium and LDH in slice cultures) in each sample.

### Preparation of slice culture lysates for western blotting

After treatments, medium was removed and slice cultures were washed in ice-cold phosphate-buffered saline (PBS), followed by lysis in extra strong lysis buffer (10 mM Tris-HCl (pH 7.5), 0.5% (w/v) sodium dodecyl sulphate (SDS), 20 mM sodium deoxycholate, 1% (v/v) Triton-X-100, 75 mM sodium chloride, 10 mM ethylenediaminetetraacetic acid (EDTA), 2 mM sodium orthovanadate, 1.25 mM sodium fluoride, and Complete protease inhibitor cocktail (Roche Diagnostics, UK)), and centrifugation at 16,000 g_av_ for 20 min at 4 °C. The protein concentration of supernatants was measured using a BCA protein assay kit (Pierce Endogen, Rockford, USA) and samples were standardised to equal protein concentration before being analysed by SDS-PAGE.

### Preparation of synaptosomes

Synaptosomes were prepared as previously described^16, 56^. In brief, slices were homogenised in synaptosome lysis buffer (10 mM Tris HCl, pH 7.4, containing 0.32 M sucrose, 2 mM EGTA, 2 mM EDTA and Complete protease inhibitor cocktail [Roche, Mannheim, Germany]) and centrifuged at 1,000 g(av) for 10 minutes at 4 °C to remove cell nuclei and debris. The supernatant (“total fraction”) was then centrifuged at 10,000 g(av) for 20 minutes at 4 °C. The final pellet (“synaptosomes”) was resuspended in 2x Laemmli buffer before immunoblotting for pre- and post-synaptic markers.

### Aβ ELISA

Quantification of Aβ1-40 and Aβ1-42 in slice lysates was performed using ELISA kits from Invitrogen (Aβ1-40 ELISA KHB3481; Aβ1-42 ELISA KHB3442) as we have previously described^36^. Aβ1-40 and Aβ1-42 standards of known concentration were plated to generate a standard curve from which Aβ content was measured. Absorbance was read at 450 nm.

### SDS-PAGE and Immunoblotting

5–20 µg protein was separated on 10% (w/v) SDS-PAGE gels and electrophoretically transferred to nitrocellulose membrane. After blocking with 5% (w/v) non-fat dried milk for 1 hour, membranes were probed with primary antibodies, followed by fluorophore-coupled secondary antibodies. Detected proteins were visualised and quantified using an Odyssey infrared imaging and analysis system (Li-Cor Biosciences, Cambridge, UK). This method enables sensitive and robust detection of protein amounts across a wide quantitative linear range^57^, in contrast to semi-quantitative chemiluminescence methods. Importantly, saturated signals are indicated when scanning and these cannot be quantified. The following primary antibodies were used for western blotting; total tau (rabbit IgG; Dako Ltd., Ely, UK); APP (mouse IgG1, clone 22c11; Millipore UK Ltd; Watford, UK), total GSK-3α/β (Mouse IgG2b, Clone 1H8, Enzo Life Sciences, Exeter, UK), GSK-3α/β-pSer21/9 (rabbit IgG, New England Biolabs, Hitchin, UK), pan-Akt (mouse IgG, Cell Signaling, Beverly, MA), Akt-pSer473 (rabbit IgG, Cell Signaling, Beverly, MA), β-catenin (mouse IgG, Abcam, Cambridge, UK), β-catenin-pSer552 (Sigma, Poole, Dorset, UK), Cdk5 (mouse IgG1, clone J-3; Santa Cruz Biotechnology, Santa Cruz, USA), p35 (rabbit IgG, clone C-19, Santa Cruz Biotechnology, Santa Cruz, USA), synaptophysin (mouse IgM, clone SP15; Enzo Life Sciences, Exeter, UK), PSD-95 (rabbit IgG, Cell Signaling, USA), β-actin (mouse IgG1, clone AC-15, Abcam, Cambridge, UK). The following tau antibodies were kindly gifted by Peter Davies (Albert Einstein College of Medicine, Bronx, NY, USA): CP13 (phospho-Ser-202; mouse IgG1), PHF-1 (phospho-Ser-396/404; mouse IgG1), Tg3 (phospho-Thr-231; mouse IgM)^58, 59^.

### Immunofluorescent staining

Organotypic brain slice cultures were fixed on their membrane inserts in 4% PFA for 4 h and stained according to Gogolla *et al*.^60^. In brief, slice cultures were cut out whilst still on their membranes and then treated as free-floating sections. Slice cultures were permeabilised for 18 h in 0.5% Triton X-100 at 4 °C and then blocked in 20% bovine serum albumin (BSA) for 4 h at room temperature (RT). Slice cultures were incubated in total tau and CP13 (as above) primary antibodies overnight at 4 °C in 5% BSA, washed and then incubated in fluorophore-coupled secondary antibodies for 4 h at RT. Slice cultures were washed a final time before mounting on slides with fluorescent mounting medium (Dako Ltd., Ely, UK). Slice cultures were imaged using an Eclipse Ti-E Inverted (Nikon Instruments, UK) microscope using a CSU-X1 Spinning Disk Confocal and Andor Ixon3 EM-CCD camera imaging system setup using a 60 Plan Apo VC N2 objective lens (Nikon Instruments, UK).

### Statistics

Data were analysed using either Student’s unpaired t-test or one-way analysis of variance (Graphpad Prism 6.0 Software), followed by Dunnett’s post-hoc tests when data was determined to follow the normal distribution according to the Shapiro-Wilk test. If data were not normally distributed, non-parametric tests (Mann Whitney U test, Kruskal-Wallis test) were performed. Differences were considered statistically significant when p < 0.05. For all slice culture experiments, N refers to the number of wells, each of which contain three brain slices. For N = 9, this is based on 3 wells from 3 independent experiments.

## Electronic supplementary material

Supplementary figure 1

Dataset 1
