# Supplementary material for: Inhibition of glycogen synthase kinase-3 by BTA-EG4 reduces tau abnormalities in an organotypic brain slice culture model of Alzheimer’s disease
Source: Sci Rep. 2017 Aug 7;7:7434. doi: 10.1038/s41598-017-07906-1 (PMC5547074; doi:10.1038/s41598-017-07906-1)
Supplement: Supplementary file 1 — Supplementary figure 1 [file 41598_2017_7906_MOESM1_ESM.pdf]

Title: Inhibition of glycogen synthase kinase-3 by BTA-EG_4 reduces tau abnormalities in an organotypic brain slice culture model of Alzheimer's disease

Authors: Cara L. Croft[1Ψ], Ksenia Kurbatskaya[1], Diane P. Hanger[1], Wendy Noble[1*]

Affiliations: [1]King's College London, Institute of Psychiatry, Psychology & Neuroscience, Maurice Wohl Clinical Neuroscience Institute, Department of Basic and Clinical Neuroscience, London, SE5 9RX. UK.
[Ψ]Current address: Department of Neuroscience, University of Florida, Gainesville, Florida, 32610, USA

Supplementary Materials and Methods

Primary cortical cell culture and treatments:

Primary cortical neuronal cultures were prepared from embryonic day 18 (E18) rat embryos as described previously (Atherton et al., 2014), plated onto glass coverslips in 12 well plates (125,000 cells/well) and maintained in culture for 7-10DIV dependent on treatment paradigm. For LiCl treatments, 10DIV primary cortical neurons were treated for 4 h with 1, 5, 10, 20, 40 mM LiCl before assessment of cell viability. 20 mM NaCl was used as vehicle. For NAPVSIPQ treatments, 7 DIV primary cortical neurons were treated for 24 h with 1 x 10^{-15}, 10^{-13}, 10^{-11}, 10^{-9}, 10^{-7} M NAPVSIPQ or vehicle (ultrapure H_2O) before assessing cell viability. For BTA-EG_4 treatments, 7 DIV primary cortical neurons were treated for 24 h with 20, 40, 60 µM BTA-EG_4 or vehicle (DMSO) before assessing cell viability. N represents 1 well of a 12-well plate treated with drug or control, n=9 for all.

Cell death assays:

After treatment, culture medium was removed and neurons washed in pre-warmed PBS. Cell death was confirmed using a live/dead fixable cell stain (Invitrogen, Paisley, UK), performed according to the manufacturer's instructions. Cell death was quantified as a proportion of total cell death (cell dye uptake).

Supp. Fig. 1

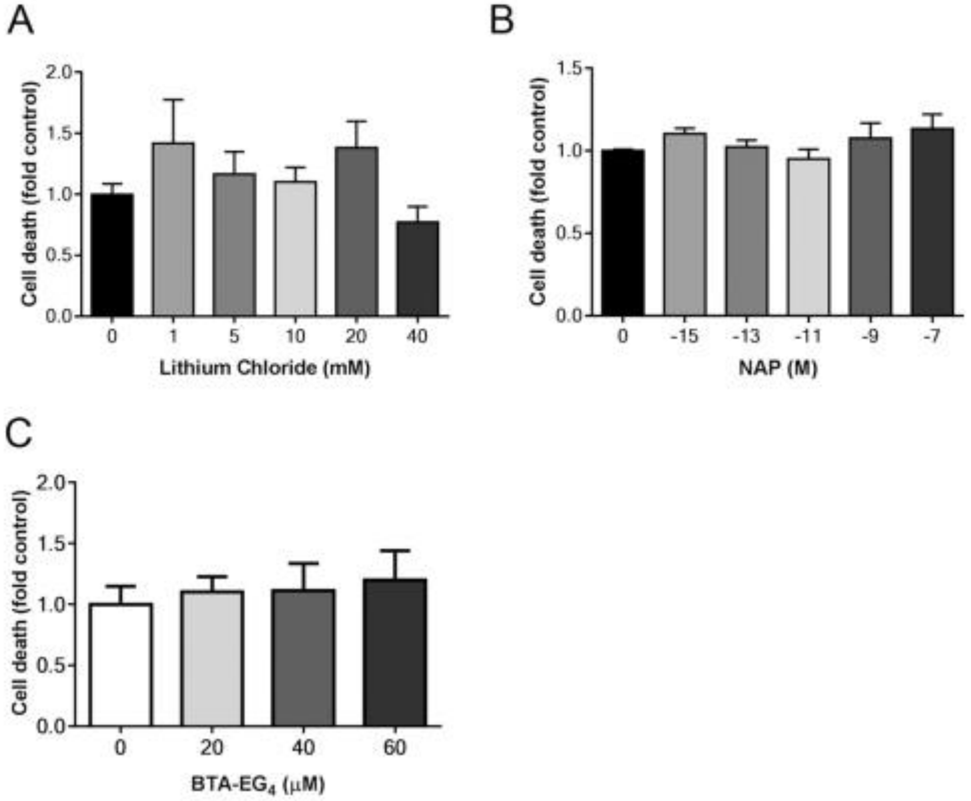

Supplementary Figure 1: LiCl, NAP, and BTA-EG$_4$ effects on cell viability. Bar charts show levels of cell death, measured by incorporation of dead cell dye, following (A) treatment of 10 DIV primary cortical cultures with 1-40mM LiCl or control (20mM NaCl) for 4h, (B) treatment of 7 DIV primary cortical cultures with 1 x 10_{-15} – 1 x 10_{-7}M NAP treatment or control (H_2O) for 24h, (C) treatment of 7 DIV primary cortical cultures for 24h with control (DMSO, 0µM), 20, 40 or 60 µM BTA-EG$_4$. Data is shown as fold change from control. Data is mean ± SEM, (n=9 for all). All data were statistically compared by one-way ANOVA.
